# Alcohol Hangover: Mechanisms and Mediators

**Published:** 1998

**Authors:** Robert Swift, Dena Davidson

**Affiliations:** Robert Swift, M.D., Ph.D., is associate professor in the Department of Psychiatry and Human Behavior at Brown University, Providence, Rhode Island, and associate chief of staff for research and education at Providence Veterans Affairs Medical Center. Dena Davidson, Ph.D., is assistant professor of psychiatry at Indiana University of Medicine, Indianapolis, Indiana

**Keywords:** post AOD intoxication state, symptom, urinalysis, gastrointestinal disorder, hypoglycemia, sleep disorder, circadian rhythm, ethanol metabolite, disorder of fluid or electrolyte or acid-base balance, nutrient intake, headache, vomiting, neurotransmitter receptors, congenors, multiple drug use, personality trait, family AODU (alcohol and other drug use) history, drug therapy, literature review

## Abstract

Hangovers are a frequent, though unpleasant, experience among people who drink to intoxication. Despite the prevalence of hangovers, however, this condition is not well understood scientifically. Multiple possible contributors to the hangover state have been investigated, and researchers have produced evidence that alcohol can directly promote hangover symptoms through its effects on urine production, the gastrointestinal tract, blood sugar concentrations, sleep patterns, and biological rhythms. In addition, researchers postulate that effects related to alcohol’s absence after a drinking bout (i.e., withdrawal), alcohol metabolism, and other factors (e.g., biologically active, nonalcohol compounds in beverages; the use of other drugs; certain personality traits; and a family history of alcoholism) also may contribute to the hangover condition. Few of the treatments commonly described for hangover have undergone scientific evaluation.

***“My first return of sense or recollection was upon waking in a strange, dismal-looking room, my head aching horridly, pains of a violent nature in every limb, and deadly sickness at the stomach. From the latter I was in some degree relieved by a very copious vomiting. Getting out of bed, I looked out of the only window in the room, but saw nothing but the backs of old houses, from which various miserable emblems of poverty were displayed . . . . At that moment I do not believe in the world there existed a more wretched creature than myself. I passed some moments in a state little short of despair . . . .” —William Hickey (Spenser 1913)***

The British writer William Hickey wrote these words in the year 1768, vividly describing the aftermath of a bout of heavy alcohol drinking—an experience commonly referred to as a “hangover.” Similar descriptions of hangovers appear in the writings of ancient Egypt and Greece as well as in the Old Testament. No doubt, prehistoric people also experienced hangovers soon after they discovered alcohol.

Despite its long history, however, hangover has received relatively scant formal attention from researchers. Little is known about the physiology underlying the hangover condition. For example, it is unclear whether hangover signs and symptoms are attributable to alcohol’s direct effects on the body, its aftereffects, or a combination of both. Similarly, investigators are uncertain about the degree to which hangover affects a person’s thinking and mentally controlled motor functions, a question with serious implications for activities such as job performance and driving. In addition, researchers know little about hangover prevention and treatment. Although folk remedies for hangovers abound, their efficacy in reducing the intensity and duration of a hangover has not received systematic study. In fact, some researchers and clinicians question whether finding an effective treatment for hangovers is desirable, given that the hangover experience may deter some people from engaging in subsequent episodes of heavy drinking.

Although gaps clearly remain in scientific knowledge about hangovers, research has elucidated several aspects. This article describes what is known about the hangover condition, the possible physiological factors contributing to it, and treatment options.

## What is A Hangover?

A hangover is characterized by the constellation of unpleasant physical and mental symptoms that occur after a bout of heavy alcohol drinking (see table 1). Physical symptoms of a hangover include fatigue, headache, increased sensitivity to light and sound, redness of the eyes, muscle aches, and thirst. Signs of increased sympathetic nervous system activity can accompany a hangover, including increased systolic blood pressure, rapid heartbeat (i.e., tachycardia), tremor, and sweating. Mental symptoms include dizziness; a sense of the room spinning (i.e., vertigo); and possible cognitive and mood disturbances, especially depression, anxiety, and irritability. The particular set of symptoms experienced and their intensity may vary from person to person and from occasion to occasion. In addition, hangover characteristics may depend on the type of alcoholic beverage consumed and the amount a person drinks. Typically, a hangover begins within several hours after the cessation of drinking, when a person’s blood alcohol concentration (BAC) is falling. Symptoms usually peak about the time BAC is zero and may continue for up to 24 hours thereafter.

Overlap exists between hangover and the symptoms of mild alcohol withdrawal (AW), leading to the assertion that hangover is a manifestation of mild withdrawal. Hangovers, however, may occur after a single bout of drinking, whereas withdrawal occurs usually after multiple, repeated bouts. Other differences between hangover and AW include a shorter period of impairment (i.e., hours for hangover versus several days for withdrawal) and a lack of hallucinations and seizures in hangover.

People experiencing a hangover feel ill and impaired. Although a hangover may impair task performance and thereby increase the risk of injury, equivocal data exist on whether hangover actually impairs complex mental tasks. When subjects with a BAC of zero were tested following alcohol intoxication with peak BAC’s in the range of 50 to 100 milligrams per deciliter (mg/dL), most of them did not show significant impairments in the performance of simple mental tasks, such as reaction time (Lemon et al. 1993). Similarly, several studies that investigated the hangover effects on a more complex mental task (i.e., simulated automobile driving) did not report impaired performance (Streufert et al. 1995; Tornros and Laurell 1991). In contrast, a study of military pilots completing a simulated flying task revealed significant decrements in some performance measures (particularly among older pilots) 8 to 14 hours after they had consumed enough alcohol to be considered legally drunk (Yesavage and Leirer 1986).

## Prevalence of Hangover

Generally, the greater the amount and duration of alcohol consumption, the more prevalent is the hangover, although some people report experiencing a hangover after drinking low levels of alcohol (i.e., one to three alcoholic drinks), and some heavy drinkers do not report experiencing hangovers at all. A survey by Harburg and colleagues (1993) on the prevalence of hangovers found that approximately 75 percent of the subjects who drank to intoxication reported experiencing a hangover at least some of the time. In a study of 2,160 Finnish men, researchers found an association between increased weekly alcohol consumption and the frequency of hangover: 43.8 percent of the group of heaviest drinkers (i.e., study subjects who drank more than 106 grams [g] of alcohol per week or approximately 9 drinks) reported experiencing a hangover monthly or more often, compared with 6.6 percent of the remaining study subjects (Kauhanen et al. 1997). Similarly, in a study of 1,041 drinkers in New York State, 50 percent of the subjects who drank two or more drinks per day reported experiencing hangovers in the previous year, whereas subjects who consumed lower levels of alcohol reported fewer hangovers (Smith and Barnes 1983). Other reports, however, claim that hangovers occur less often in heavy drinkers. In a study of 43 alcoholic drinkers admitted for inpatient treatment, 50 percent of the subjects reported experiencing no hangovers within the previous year and 23 percent reported never experiencing a hangover (Pristach et al. 1983).

## Physiological Factors Contributing to Hangover

Hangover symptoms have been attributed to several causes (see table 2), including the direct physiological effects of alcohol on the brain and other organs; the effects of the removal of alcohol from these organs after alcohol exposure (i.e., withdrawal); the physiological effects of compounds produced as a result of alcohol’s metabolism (i.e., metabolites), especially acetaldehyde; and nonalcohol factors, such as the toxic effects of other biologically active chemicals (i.e., congeners) in the beverage, behaviors associated with the alcohol-drinking bout (e.g., other drug use, restricted food intake, and disruption of normal sleep time), and certain personal characteristics (e.g., temperament, personality, and family history of alcoholism). Although current evidence suggests that more than one factor most likely contributes to the overall hangover state, the following sections address each of the postulated causes in turn.

### Direct Alcohol Effects

Alcohol may directly contribute to a hangover in several ways, including the following.

#### Dehydration and Electrolyte Imbalance

Alcohol causes the body to increase urinary output (i.e., it is a diuretic). The consumption of 50 g of alcohol in 250 milliliters (mL) of water (i.e. approximately 4 drinks) causes the elimination of 600 to 1,000 mL (or up to 1 quart) of water over several hours (Montastruc 1986). Alcohol promotes urine production by inhibiting the release of a hormone (i.e., antidiuretic hormone, or vasopressin) from the pituitary gland. In turn, reduced levels of antidiuretic hormone prevent the kidneys from reabsorbing (i.e., conserving) water and thereby increase urine production. Additional mechanisms must be at work to increase urine production, however, because antidiuretic hormone levels increase as BAC levels decline to zero during hangover (Eisenhofer et al. 1985). Sweating, vomiting, and diarrhea also commonly occur during a hangover, and these conditions can result in additional fluid loss and electrolyte imbalances. Symptoms of mild to moderate dehydration include thirst, weakness, dryness of mucous membranes, dizziness, and lightheadedness— all commonly observed during a hangover.

#### Gastrointestinal Disturbances

Alcohol directly irritates the stomach and intestines, causing inflammation of the stomach lining (i.e., gastritis) and delayed stomach emptying, especially when beverages with a high alcohol concentration (i.e., greater than 15 percent) are consumed (Lieber 1995). High levels of alcohol consumption also can produce fatty liver, an accumulation of fat compounds called triglycerides and their components (i.e., free fatty acids) in liver cells. In addition, alcohol increases the production of gastric acid as well as pancreatic and intestinal secretions. Any or all of these factors can result in the upper abdominal pain, nausea, and vomiting experienced during a hangover.

#### Low Blood Sugar

Several alterations in the metabolic state of the liver and other organs occur in response to the presence of alcohol in the body and can result in low blood sugar levels (i.e., low glucose levels, or hypoglycemia) (National Institute on Alcohol Abuse and Alcoholism 1994). Alcohol metabolism leads to fatty liver (described earlier) and a buildup of an intermediate metabolic product, lactic acid, in body fluids (i.e., lactic acidosis). Both of these effects can inhibit glucose production.

Alcohol-induced hypoglycemia generally occurs after binge drinking over several days in alcoholics who have not been eating. In such a situation, prolonged alcohol consumption, coupled with poor nutritional intake, not only decreases glucose production but also exhausts the reserves of glucose stored in the liver in the form of glycogen, thereby leading to hypoglycemia. Because glucose is the primary energy source of the brain, hypoglycemia can contribute to hangover symptoms such as fatigue, weakness, and mood disturbances. Diabetics are particularly sensitive to the alcohol-induced alterations in blood glucose. However, it has not been documented whether low blood sugar concentrations contribute to hangover symptomatically.

#### Disruption of Sleep and Other Biological Rhythms

Although alcohol has sedative effects that can promote sleep onset, the fatigue experienced during a hangover results from alcohol’s disruptive effects on sleep. Alcohol-induced sleep may be of shorter duration and poorer quality because of rebound excitation (see the section “Effects of Alcohol Withdrawal”) after BAC’s fall, leading to insomnia (Walsh et al. 1991). Furthermore, when drinking behavior takes place in the evening or at night (as it often does), it can compete with sleep time, thereby reducing the length of time a person sleeps. Alcohol also disrupts the normal sleep pattern, decreasing the time spent in the dreaming state (i.e., rapid eye movement [REM] sleep) and increasing the time spent in deep (i.e., slow-wave) sleep. In addition, alcohol relaxes the throat muscles, resulting in increased snoring and, possibly, periodic cessation of breathing (i.e., sleep apnea).

Alcohol interferes with other biological rhythms as well, and these effects persist into the hangover period. For example, alcohol disrupts the normal 24-hour (i.e., circadian) rhythm in body temperature, inducing a body temperature that is abnormally low during intoxication and abnormally high during a hangover. Alcohol intoxication also interferes with the circadian nighttime secretion of growth hormone, which is important in bone growth and protein synthesis. In contrast, alcohol induces the release of adrenocorticotropic hormone from the pituitary gland, which in turn stimulates the release of cortisol, a hormone that plays a role in carbohydrate metabolism and stress response; alcohol thereby disrupts the normal circadian rise and fall of cortisol levels. Overall, alcohol’s disruption of circadian rhythms induces a “jet lag” that is hypothesized to account for some of the deleterious effects of a hangover (Gauvin et al. 1997).

### Alcohol and Headache

In a large epidemiological survey of headache in Danish 25- to 64-year-olds, the lifetime prevalence of hangover headache was 72 percent, making it the most common type of headache reported (Rasmussen and Olesen 1992). Alcohol intoxication results in vasodilatation, which may induce headaches. Alcohol has effects on several neurotransmitters and hormones that are implicated in the pathogenesis of headaches, including histamine, serotonin, and prostaglandins (Parantainen 1983). However, the etiology of hangover headache remains unknown.

### Effects of Alcohol Withdrawal

The AW syndrome following the cessation of excessive drinking results from compensatory changes in the central nervous system that take place in response to chronically administered depressant substances (in this case, alcohol, or more specifically, ethanol). These changes include alterations in two types of receptors embedded in nerve cell membranes. One receptor type binds with an important chemical messenger (i.e., neurotransmitter) called gamma-aminobutyric acid (GABA), and the other type binds with another neurotransmitter, glutamate. Both GABA and glutamate are critical in regulating nerve cell activity: GABA is the body’s primary means of inhibiting nerve cell activity, and glutamate is the primary means of exciting it.

Following chronic alcohol exposure, the body decreases (i.e., downregulates) the number or sensitivity of GABA receptors and increases (i.e., upregulates) the number or sensitivity of glutamate receptors in an effort to counterbalance alcohol’s sedative effects. When alcohol is removed from the body, however, the central nervous system and the portion of the nervous system that coordinates response to stress (i.e., the sympathetic nervous system) remain in an unbalanced “overdrive” state (Tsai et al. 1995). Sympathetic nervous system hyperactivity accounts for the tremors, sweating, and tachycardia observed in both hangover and AW syndrome.

Several lines of evidence suggest that a hangover is a mild manifestation of the AW syndrome in non-alcohol-dependent drinkers. First, the signs and symptoms of hangover and mild AW overlap considerably. The revised Clinical Institute Withdrawal Assessment for Alcohol (CIWA-Ar) scale, an instrument widely used to assess the severity of a withdrawal episode in alcohol-dependent patients, measures 10 withdrawal-associated items: nausea and vomiting; tremor; sweating; anxiety; agitation; headache; disturbances in the sense of touch, hearing, and vision (e.g., hallucinations); and orientation (e.g., awareness of the date and location) (Sullivan et al. 1989, see also p. 8 of the article by Saitz for a sample of the assessment form). Several of these items also are usually present during a hangover, including nausea and vomiting, tremor, sweating, anxiety, headache, and sensory disturbances.

Second, Begleiter and colleagues (1974) present evidence that the hangover condition is actually a state of central nervous system excitation, despite the perceived sedation and malaise. Support for this view comes from the research of Pinel and Mucha (1980), which shows that single doses of alcohol decrease seizure thresholds in animals several hours later. Their finding indicates rebound excitation, a phenomenon noted to occur after short-term administration of some sedatives that can quickly clear the body, including alcohol and certain benzodiazepine drugs.

Third, the observation that alcohol readministration alleviates the unpleasantness of both AW syndrome and hangovers suggests that the two experiences share a common process.

### Effects of Alcohol Metabolites

Alcohol undergoes a two-step process in its metabolism (see figure). First, an enzyme (i.e., alcohol dehydrogenase) metabolizes alcohol to an intermediate product, acetaldehyde; then a second enzyme (aldehyde dehydrogenase [ALDH]) metabolizes acetaldehyde to acetate. Acetaldehyde is a chemically reactive substance that binds to proteins and other biologically important compounds. At higher concentrations, it causes toxic effects, such as a rapid pulse, sweating, skin flushing, nausea, and vomiting. In most people, ALDH metabolizes acetaldehyde quickly and efficiently, so that this intermediate metabolite does not accumulate in high concentrations, although small amounts are present in the blood during alcohol intoxication. In some people, however, genetic variants of the ALDH enzyme permit acetaldehyde to accumulate. Those people routinely flush, sweat, and become ill after consuming small amounts of alcohol.

Because of the similarity between the acetaldehyde reaction and a hangover, some investigators have suggested that acetaldehyde causes hangovers. Although free acetaldehyde is not present in the blood after BAC’s reach zero, the toxic effects of acetaldehyde produced during alcohol metabolism may persist into the hangover period.

### Effects of Factors Other Than Alcohol

Factors other than alcohol also may contribute to a hangover. These factors include the following possibilities.

#### Congeners

Among other reasons, people consume alcoholic beverages for their ethanol content. Most alcoholic beverages contain smaller amounts of other biologically active compounds, however, including other alcohols. These compounds, known as congeners, contribute to the taste, smell, and appearance of alcoholic beverages. Congeners may be produced along with ethanol during fermentation, generated during aging or processing through the degradation of the beverage’s organic components, or added to the beverage during the production process. Investigators now believe that congeners may contribute to a beverage’s intoxicating effects and to a subsequent hangover. Research has shown that beverages composed of more pure ethanol, such as gin or vodka, induce fewer hangover effects than do beverages containing a large number of congeners, such as whiskey, brandy, or red wine (Chapman 1970; Pawan 1973). A hangover also may occur when pure ethanol is administered, however.

One specific congener implicated in hangover effects is methanol, which is an alcohol compound found in alcoholic beverages along with ethanol. The two compounds differ slightly in chemical structure in that methanol contains one less carbon atom and two fewer hydrogen atoms than ethanol. The same enzymes that metabolize ethanol, alcohol dehydrogenase, and aldehyde dehydrogenase also metabolize methanol; however, the products of methanol metabolism (i.e., formaldehyde and formic acid) are extremely toxic and in high concentrations may cause blindness and death.

Support for methanol’s contribution to hangovers comes from several sources. For example, distilled spirits that are more frequently associated with the development of a hangover, such as brandies and whiskeys, contain the highest concentrations of methanol. Moreover, in an experimental study with four subjects who consumed red wine containing 100 milligrams per liter (mg/L) of methanol, Jones (1987) found that elevated blood levels of methanol persisted for several hours after ethanol was metabolized, which corresponded to the time course of hangover symptoms. Methanol lingers after ethanol levels drop, because ethanol competitively inhibits methanol metabolism. The fact that ethanol readministration fends off hangover effects may be further evidence of methanol’s contribution to the hangover condition, given ethanol’s ability to block methanol metabolism and thereby slow the production of formaldehyde and formic acid.

Certain people develop headaches soon after drinking red wine but not after drinking white wine or vodka. Recent research finds that red wine, but not white wine or vodka, can increase plasma serotonin and plasma histamine levels. The specific agents in wine responsible for these increased levels are not known. Increased plasma serotonin and histamine can trigger headaches in susceptible people (Pattichis et al. 1995; Jarisch and Wantke 1996).

#### Use of Other Drugs

The use of other drugs often accompanies heavy alcohol consumption. Most heavy drinkers smoke cigarettes, and some also use marijuana, cocaine, or other drugs. Although certain drugs can themselves produce hangover symptoms and affect alcohol intoxication, the effects of the various alcohol and other drug combinations on alcohol hangover are unknown.

**Figure:**
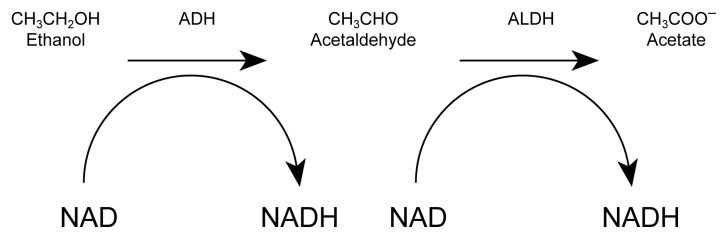
The metabolism of beverage alcohol (i.e., ethanol) by the alcohol dehydrogenase (ADH) pathway. NOTE: ADH = alcohol dehydrogenase; ALDH = aldehyde dehydrogenase; NAD = nicotinamide adenine dinucleotide; NADH = reduced NAD.

#### Personal Influences

Some evidence exists that increased hangover symptoms occur more often in people possessing certain personality traits, such as neuroticism, anger, and defensiveness. Negative life events and feelings of guilt about drinking also are associated with experiencing more hangovers (Harburg et al. 1993). In addition, Earleywine (1993*a*,*b*) reports greater hangover symptoms in people who have a higher personality risk for the development of alcoholism (as measured by the Mac-Andrew Scale [MacAndrew 1965]). Those studies suggest that people who have an elevated personality risk for alcoholism experience more acute withdrawal and hangover symptoms and may initiate further drinking in an effort to find relief.

Research has shown that a history of alcoholism in a person’s family (i.e., a positive family history) is associated with a decreased sensitivity to the intoxicating effects of alcohol and a greater risk for developing alcoholism (Schuckit and Smith 1996). Newlin and Pretorius (1990) suggested that a positive family history for alcoholism may be associated with a tendency for increased hangover symptoms as well. Their research compared the self-reported hangover symptoms in college-age sons of alcoholic fathers with symptoms in sons of nonalcoholic fathers and found that the subjects with a positive family history for alcoholism had had greater hangover symptoms during the previous year. The amount of drinking was comparable between the two groups, although the subjects with a positive family history reported consuming significantly more mixed drinks than the group with a negative family history.

## Treatments for Hangover

Many treatments are described to prevent hangover, shorten its duration, and reduce the severity of its symptoms, including innumerable folk remedies and recommendations. Few treatments have undergone rigorous investigation, however. Conservative management offers the best course of treatment. Time is the most important component, because hangover symptoms will usually abate over 8 to 24 hours.

Attentiveness to the quantity and quality of alcohol consumed can have a significant effect on preventing hangover. Hangover symptoms are less likely to occur if a person drinks only small, nonintoxicating amounts. Even among people who drink to intoxication, those who consume lower amounts of alcohol appear less likely to develop a hangover than those who drink higher amounts. Hangovers have not been associated with drinking beverages with a low alcohol content or with drinking nonalcoholic beverages.

The type of alcohol consumed also may have a significant effect on reducing hangover (Chapman 1970; Pawan 1973). Alcoholic beverages that contain few congeners (e.g., pure ethanol, vodka, and gin) are associated with a lower incidence of hangover than are beverages that contain a number of congeners (e.g., brandy, whiskey, and red wine).

Other interventions may reduce the intensity of a hangover but have not been systematically studied. Consumption of fruits, fruit juices, or other fructose-containing foods is reported to decrease hangover intensity, for example (Seppala et al. 1976). Also, bland foods containing complex carbohydrates, such as toast or crackers, can counter low blood sugar levels in people subject to hypoglycemia and can possibly relieve nausea. In addition, adequate sleep may ease the fatigue associated with sleep deprivation, and drinking nonalcoholic beverages during and after alcohol consumption may reduce alcohol-induced dehydration.

Certain medications may provide symptomatic relief for hangover symptoms. For example, antacids may alleviate nausea and gastritis. Aspirin and other nonsteroidal anti-inflammatory medications (e.g., ibuprofen or naproxen) may reduce the headache and muscle aches associated with a hangover but should be used cautiously, particularly if upper abdominal pain or nausea is present. Anti-inflammatory medications are themselves gastric irritants and will compound alcohol-induced gastritis. Although acetaminophen is a common alternative to aspirin, its use should be avoided during the hangover period, because alcohol metabolism enhances acetaminophen’s toxicity to the liver (Girre et al. 1993).

Propranolol, a beta-adrenergic antagonist^1^ used to treat high blood pressure and migraine headaches, reduces the sympathetic hyperactivity of AW; however, a small, double-blind, placebo-controlled study did not find propranolol to be effective in reducing hangover symptoms, including headache (Bogin et al. 1987). Antagonists at the serotonin-3 receptor,^2^ such as ondansetron and tropisetron, are antiemetics (i.e., they control nausea and vomiting) and block certain alcohol effects; however, a small clinical trial did not show efficacy in alleviating hangover (Muhonen et al. 1997). Caffeine (often taken as coffee) is commonly used to counteract the fatigue and malaise associated with the hangover condition. Although this traditional practice lacks scientific support, William Hickey, quoted at the beginning of this article, wrote that “very strong coffee proved of infinite benefit” (Spenser 1913).

Readministration of alcohol—the “hair of the dog that bit you” remedy—reportedly cures a hangover, but people experiencing a hangover should avoid further alcohol use. Additional drinking will only enhance the existing toxicity of the alcohol consumed during the previous bout and may increase the likelihood of even further drinking.

## Areas for Future Study

Several topics related to hangovers warrant research attention. The effect of congeners, especially methanol, on the occurrence of hangover needs closer examination, for example. Such research could help determine whether it is ethanol or congeners that produce the major signs and symptoms of hangover, and an answer to this key question would advance our understanding of hangover pathophysiology.

A particularly intriguing observation is that people with traits associated with an increased risk for alcoholism (e.g., certain personality factors or a positive family history for alcoholism) experience more severe hangovers. Although logic dictates that enduring more severe or more frequent hangovers would deter, rather than promote, further alcohol drinking, mild AW symptoms are also associated with an increased craving for alcohol (Littleton and Little 1994). Future investigations could focus on whether people can be biologically predisposed to experience more severe AW and whether such a tendency in turn predisposes them to increased alcohol consumption.

Animal models of hangover have been developed and may provide insights into the physiological and behavioral changes that occur in the period immediately after intoxication (Gauvin et al. 1993, 1997). These animal models could be used to explore the effects of early withdrawal and of congeners and to determine whether hangovers predispose to or deter further alcohol consumption.

In summary, hangover is a complex state that probably cannot be understood by a unitary explanation. Understanding the hangover condition, however, will lead to a better comprehension of the physiological effects of alcohol and the adaptive responses that alcohol engenders.

## Figures and Tables

**Table 1:** Symptoms of Hangover

Class of Symptoms	Type
Constitutional	Fatigue, weakness, and thirst
Pain	Headache and muscle aches
Gastrointestinal	Nausea, vomiting, and stomach pain
Sleep and biological rhythms	Decreased sleep, decreased REM,^1^ and increased slow-wave sleep
Sensory	Vertigo and sensitivity to light and sound
Cognitive	Decreased attention and concentration
Mood	Depression, anxiety, and irritability
Sympathetic hyperactivity	Tremor, sweating, and increased pulse and systolic blood pressure

1 REM = rapid eye movements.

**Table 2:** Possible Contributing Factors to Hangover

Direct effects of alcohol
Dehydration Electrolyte imbalance Gastrointestinal disturbances Low blood sugar Sleep and biological rhythm disturbances
Alcohol withdrawal
Alcohol metabolism (i.e., acetaldehyde toxicity)
Nonalcohol effects Compounds other than alcohol in beverages, especially methanol Use of other drugs, especially nicotine Personality type Family history for alcoholism

## Footnotes

1 An antagonist is an agent that interferes with or blocks the action of another agent, disease, or structure. A beta-adrenergic antagonist blocks the action of certain neurotransmitters governing the sympathetic nervous system.

2 Serotonin antagonists block the actions of serotonin, a neurotransmitter that can affect mood as well as inhibit gastric secretion and stimulate smooth muscle, such as that governing the contractions of the digestive system.
